# Fire forbids fifty-fifty forest

**DOI:** 10.1371/journal.pone.0191027

**Published:** 2018-01-19

**Authors:** Egbert H. van Nes, Arie Staal, Stijn Hantson, Milena Holmgren, Salvador Pueyo, Rafael E. Bernardi, Bernardo M. Flores, Chi Xu, Marten Scheffer

**Affiliations:** 1 Aquatic Ecology and Water Quality Management Group, Environmental Science Department, Wageningen University, Wageningen, The Netherlands; 2 Karlsruhe Institute of Technology, Institute of Meteorology and Climate Research, Atmospheric Environmental Research, Garmisch-Partenkirchen, Germany; 3 Resource Ecology Group, Wageningen University, Wageningen, The Netherlands; 4 Departament de Biologia Evolutiva, Ecologia i Ciències Ambientals, Universitat de Barcelona, Barcelona, Catalonia, Spain; 5 Centro Universitario Regional Este (CURE), Universidad de la República, Maldonado, Uruguay; 6 Ecology Department, Center for Biosciences, Federal University of Rio Grande do Norte, RN, Natal, Brazil; 7 School of Life Sciences, Nanjing University, Nanjing, China; University of Oregon, UNITED STATES

## Abstract

Recent studies have interpreted patterns of remotely sensed tree cover as evidence that forest with intermediate tree cover might be unstable in the tropics, as it will tip into either a closed forest or a more open savanna state. Here we show that across all continents the frequency of wildfires rises sharply as tree cover falls below ~40%. Using a simple empirical model, we hypothesize that the steepness of this pattern causes intermediate tree cover (30‒60%) to be unstable for a broad range of assumptions on tree growth and fire-driven mortality. We show that across all continents, observed frequency distributions of tropical tree cover are consistent with this hypothesis. We argue that percolation of fire through an open landscape may explain the remarkably universal rise of fire frequency around a critical tree cover, but we show that simple percolation models cannot predict the actual threshold quantitatively. The fire-driven instability of intermediate states implies that tree cover will not change smoothly with climate or other stressors and shifts between closed forest and a state of low tree cover will likely tend to be relatively sharp and difficult to reverse.

## Introduction

The emerging idea that tropical forest and savanna may be alternative stable states over a range of climatic conditions [1–5] has profound implications for predicting and managing change in these biomes. However, proving the existence of such alternative ecosystem states is notoriously difficult [6], especially in systems such as the tropical rainforest where the relevant spatial and temporal scales make replicated experimentation challenging [7–11]. Building a convincing case for hypotheses on such large-scale phenomena therefore has to rely on a combination of remotely sensed observations and constrained field experiments with a mechanistic understanding of key processes, brought together to analyze the coherence between these different lines of evidence [6]. The central hypothesis proposed to explain bistability of savanna and forest states is the existence of a strong feedback between tree cover and fire risk [4, 12–17]. The idea is that if tree cover becomes sufficiently dense, it precludes the growth of grasses that serve as an easily ignitable fuel for wildfires [16]. This is consistent with the observation that grass growth is largely suppressed when tree canopy density exceeds a critical value (roughly a Leaf Area Index of three [3]). Moreover, at much larger scales across both African and South American landscapes, it has been noted that the observed burned area is very small in landscapes with more than 40% tree cover [13, 18]. Such observations resonate with the idea of a positive feedback in which trees can prevent fire, thus stabilizing a forest state versus a landscape that is maintained open through fire [2, 4, 19].

Here we use remotely sensed data on fire frequencies at 500 m resolution, tropical tree cover and climatic variables to develop a simple model that we use to evaluate whether the fire feedback hypothesis is consistent with observed patterns of tree cover and fire, and present simulations that provide a mechanistic explanation of those patterns.

## Results

### Patterns of fire frequency

**Fig 1:**
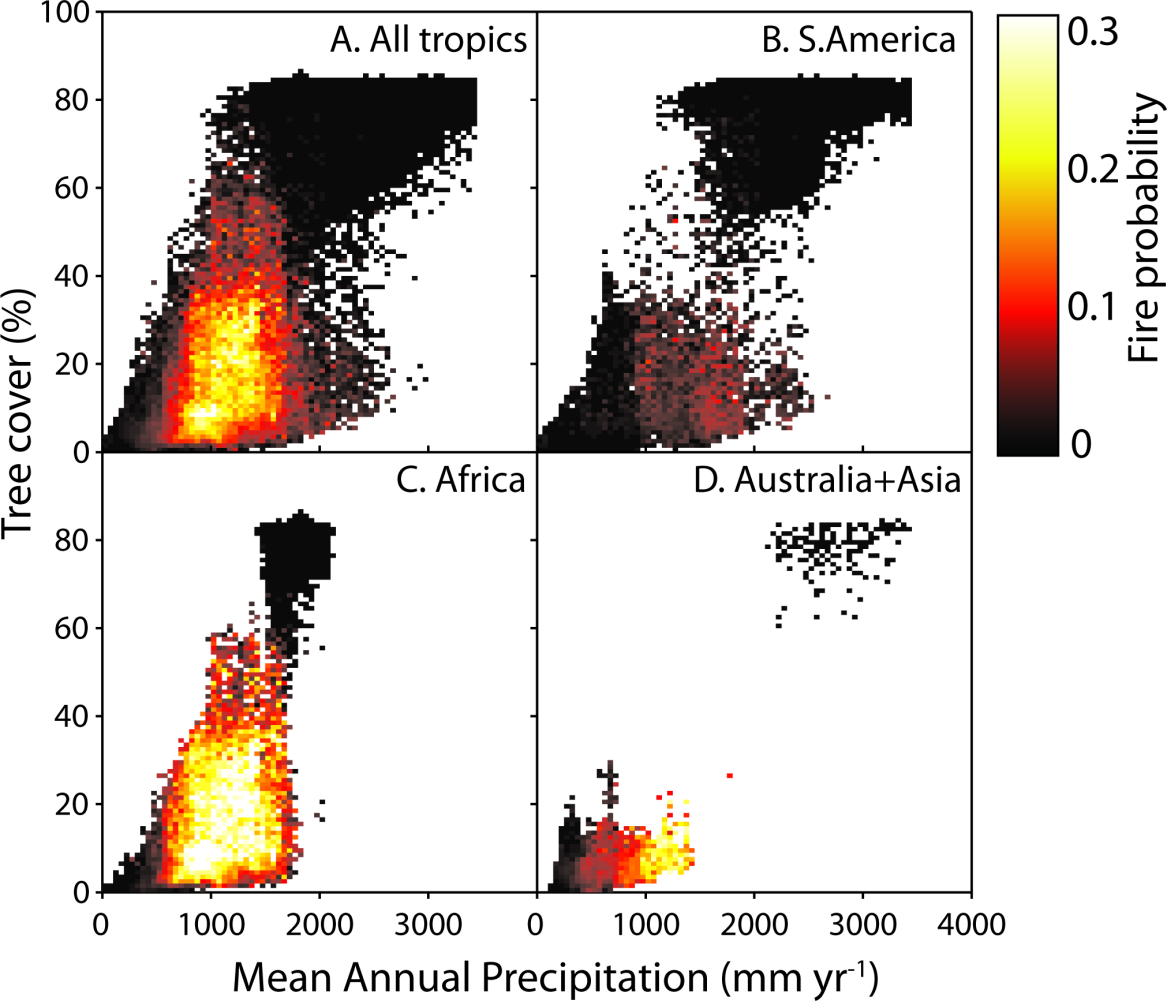
The average probability that a grid cell (500×500 m) catches fire per year as a function of mean annual precipitation and tree cover. A. All tropics, B. South America, C. Africa and D. Australia and Asia.

**Fig 2:**
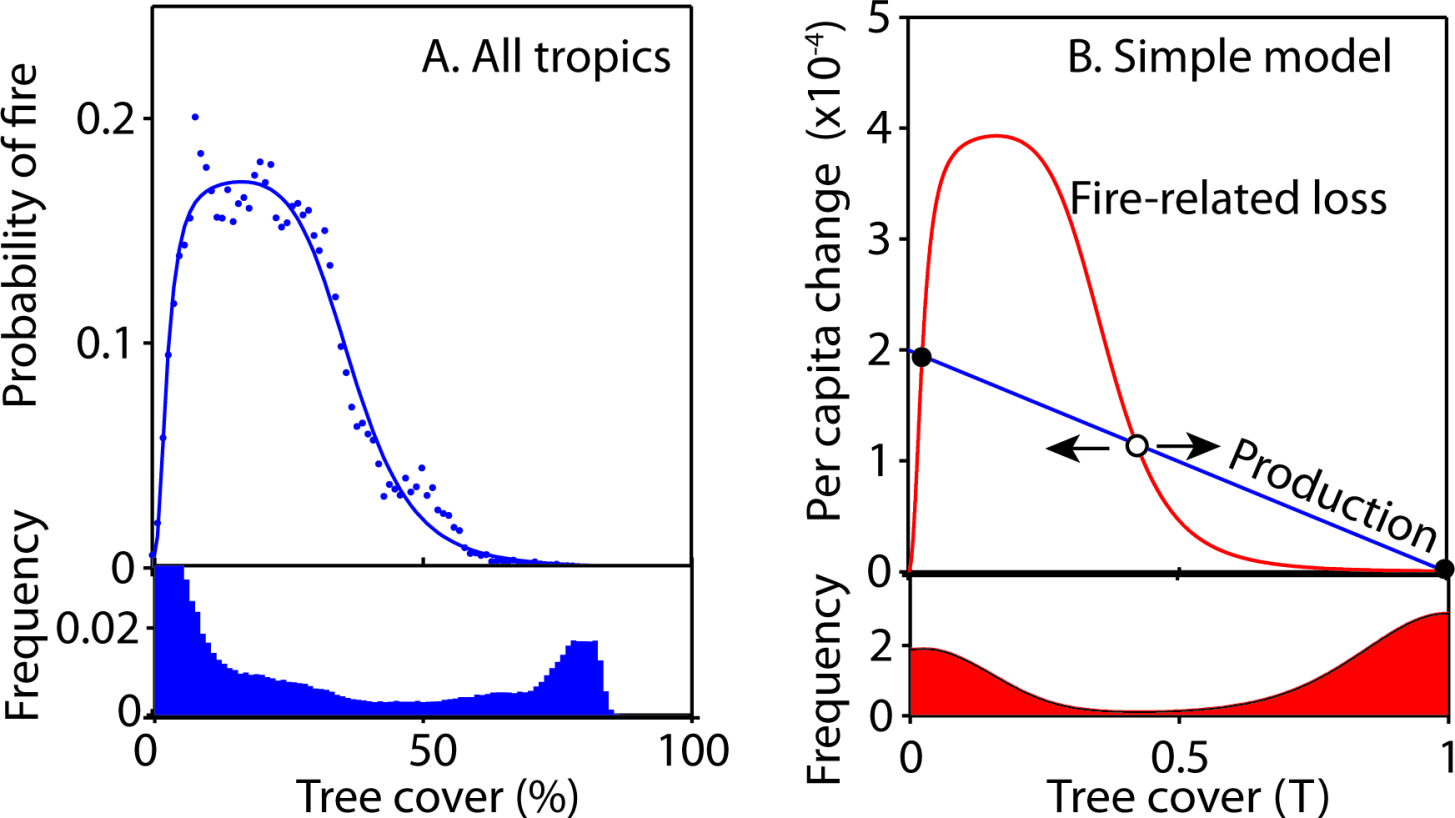
Tree-cover loss due to fire leads to alternative stable states for reasonable assumptions about the growth curve. A. The dots represent the average probability that a grid cell (500x500 m) catches fire per year as a function of tree cover (for each 1% bin). For parameters of the fitted line, see S1 Table. Below the figure the corresponding frequency of each tree cover class of 1% is given. Note that the remote sensing estimator of tree cover is bounded to maximum values just above 80%. B. If the *per-capita* growth rate (d^-1^) (blue line) is equal to the *per-capita* loss (d^-1^) due to fire (red line) the system is in equilibrium. The equilibrium at intermediate tree cover is unstable. The probability density in the lower panel is produced by exposing the model to a stochastic environment. The parameters of the model *r* = 0.0002, *α* = 0, *β* = 1, *γ* = 1, *m*_*fire*_ = 0.0004, and applying additive, normally distributed noise with a standard deviation of 0.003 using the Fokker-Planck equation ([23, 24], also see Supplementary Material) and simulating until equilibrium is reached.

**Fig 3:**
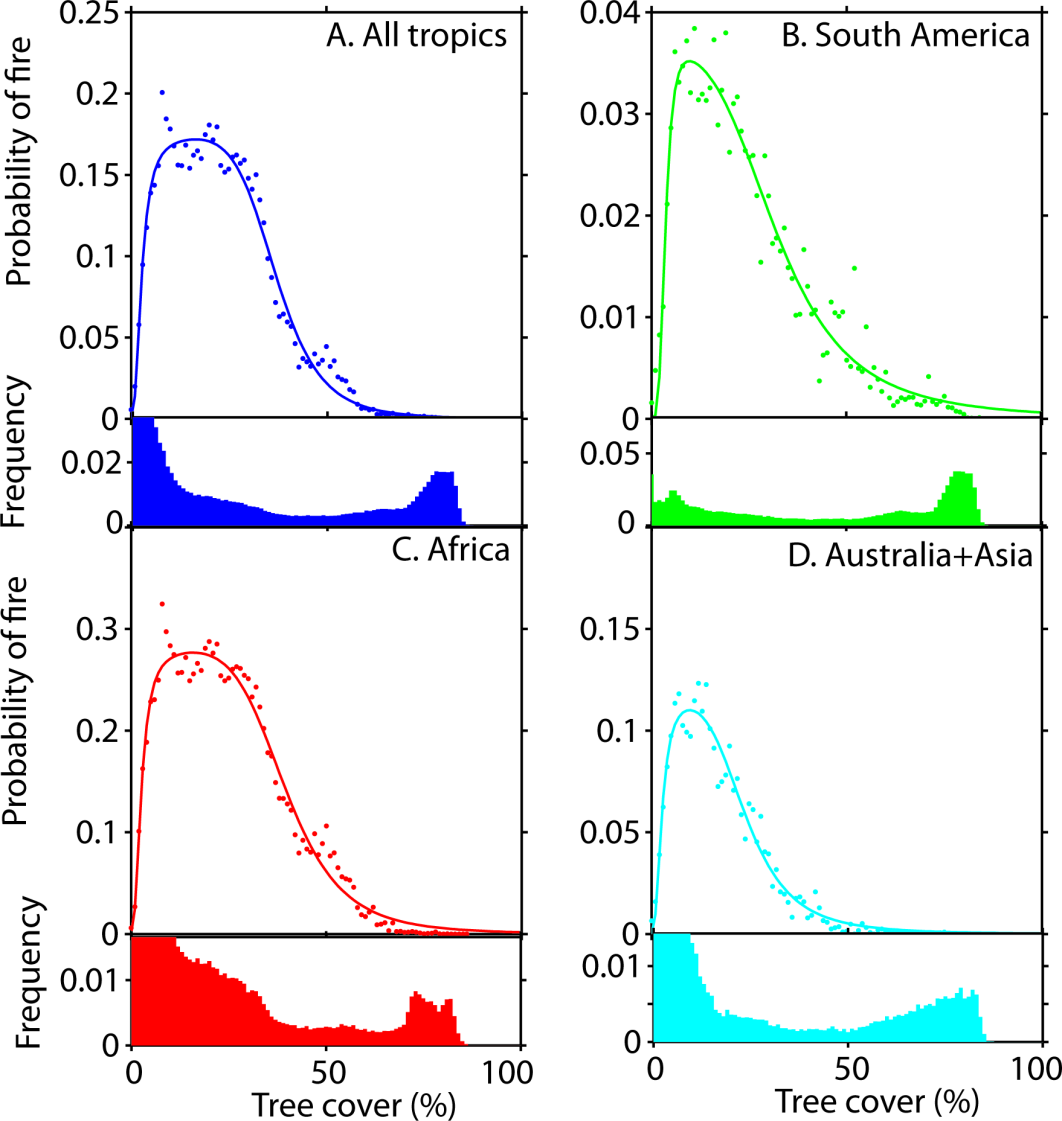
The average probability that a grid cell (500x500 m) catches fire per year as a function of tree cover. Below each figure the corresponding frequency of each tree cover class of 1% is given. A. All tropics, B. South America, C. Africa and D. Australia and Asia. Fitted line with the best AIC: $P_{fire}(T)=p1\frac{T^{p_{3}}}{T^{p_{3}}+{p_{2}}^{p_{3}}}\frac{{p_{4}}^{p5}}{T^{p_{5}}+{p_{4}}^{p5}}$. For parameters see S1 Table. See S2 Table for the precise ranges in tree cover where the fire frequency drops.

Mean annual precipitation (MAP) and tree cover explain much of the variation in fire frequency (S1 Table; S1 Fig). Our results reveal a clear and consistent rise in fire probability at a tree cover below ~40% on all continents (Figs 1, 2A and 3, S2 Table). The shape of this relationship remains rather constant across a range of classes of MAP (S2 Fig). In line with previous work [20, 21], we find that fire frequency peaks at intermediate MAP (S3 and S1C Figs), but this effect is rather independent from the effect of tree cover (Fig 1, S2 Fig). We also find substantial differences in the fire probability between continents. Especially notable is the low fire frequency in South America as compared to other continents (Figs 1 and 3, S3 Fig) and particularly Africa (see also [20, 22]). We could not explain this difference in fire frequency by any of a range of examined climatic and demographic variables (S1 Fig).

### Percolation as a potential mechanism explaining the patterns

**Fig 4:**
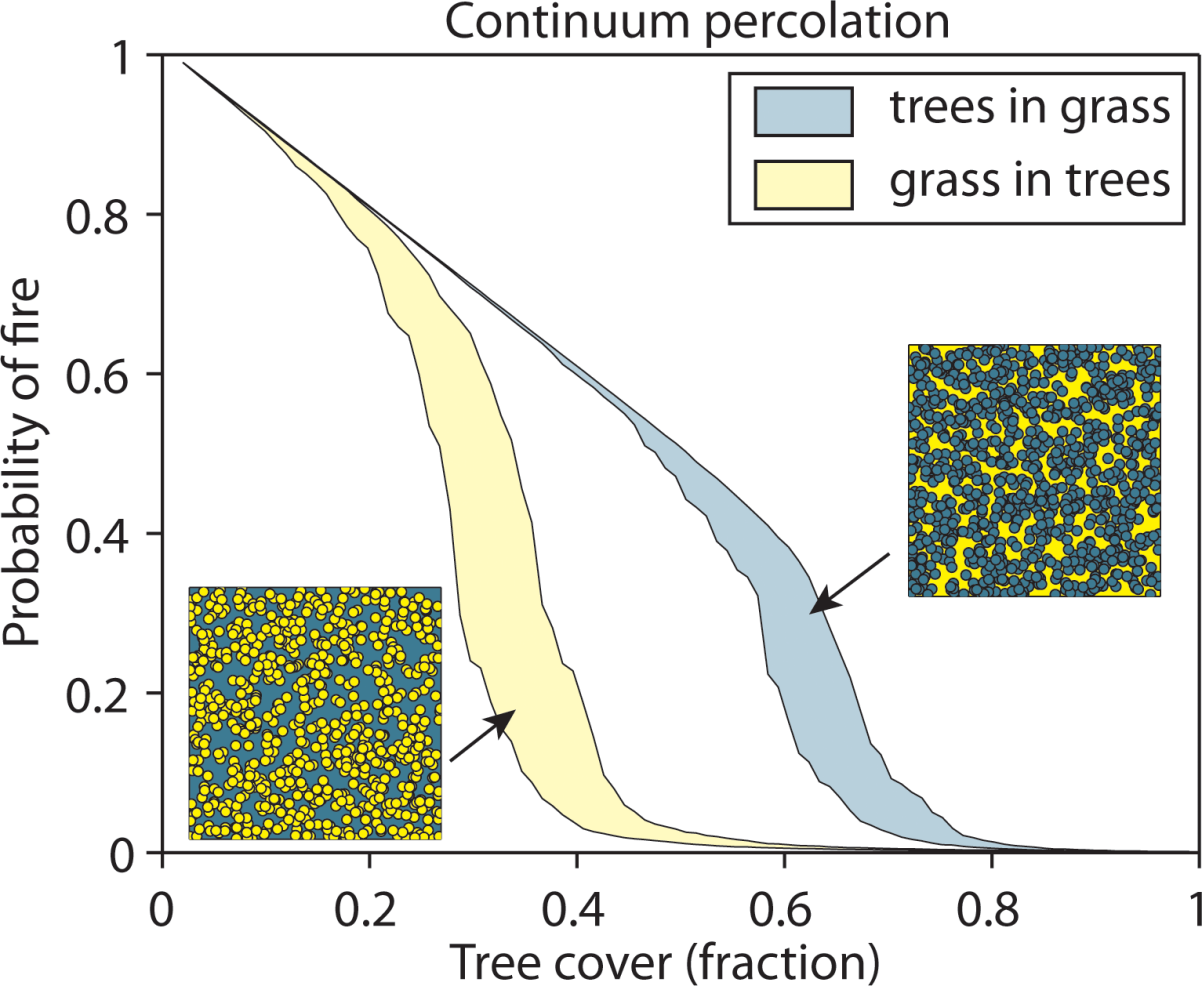
Prediction of the average probability of fire using the continuum percolation model (see Methods) with different assumptions about spatial configurations. Fire in grass spreads within connected grass areas; trees do not burn. The areas indicate the ranges between the 5^th^ and 95^th^ percentiles of the average probability of fire calculated in 100 independent runs. Yellow area: randomly dispersed overlapping circles of grass in a continuum of trees; blue area: randomly dispersed overlapping circles of trees in a continuum of grass.

The universality of the sharp drop in fire frequency above a critical tree cover is consistent with the idea that percolation might play a role in determining the impact of fire on landscapes [25–29]. The basic idea is simple: if, starting from a closed forest, tree cover decreases gradually, there will be a point when grass patches become sufficiently connected to allow fire to find a path to cross the entire landscape. This “percolation point” comes rather abruptly. Indeed, statistically, the size of the largest connected patch of grass increases sharply around a percolation point of grass cover. If we assume for simplicity that grass fires are always stopped if they run into a tree barrier, and local ignitions happen only occasionally, then the overall probability for grass to catch fire will depend on how well fire can spread through the landscape. Not surprisingly, this probability rises sharply around the percolation point. It should be noted, however, that it is not possible to predict a universal percolation point (critical tree cover) from simple models. This is because the value of the percolation threshold is strongly dependent on the spatial configuration of trees and on the connectivity between cells. For instance, if one models trees as circular patches in a continuum of grass one gets a different result than if one assumes circular open patches of grass in a continuum of trees (Fig 4). Also, if one models percolation on a lattice, the predictions depend on the connectivity between the cells, i.e. whether the cells are square, hexagonal or shaped otherwise (S4 Fig). Overall predictions of the percolation point from simple models range between 30 and 70% tree cover (Fig 4), including the 40% tree cover at which the steep change in fire frequency is observed. Clearly, the simple models do not capture other factors that will likely affect the relationship between tree density and fire frequency in reality, such as fire management and land use [30], imperfect suppression of fire by trees [31] and the fact that forest patches are neither perfectly circular nor distributed randomly. For example, in southern Africa power-law distributions of tree cluster sizes have been observed for tree cover values up to 65%, indicating effects of local-scale facilitation on tree density [32]. Nonetheless, the fact that the steep change in fire frequency around 40% tree cover is so consistent across the tropics suggests that, although we do not have sufficient information to parameterize a specific realistic model, a universal phenomenon such as percolation likely governs the relationship between tree cover and fire dynamics.

### The fire feedback hypothesis

To address the question under which conditions a drop in fire probability above a critical tree cover could cause intermediate tree cover to be unstable, resulting in alternative stable states of low and high cover, we need to consider the role of fire in the overall dynamic equilibrium of tree cover. Various modeling approaches have been developed for this, ranging from simple [33, 34] to more complex models [13, 19, 27, 35, 36]. Here we design a very simple model (Fig 2B) with the objective to give a minimalistic explanation of how the empirical patterns in fire occurrence could lead to alternative stable states. We assume that: 1) the relative loss of tree cover increases monotonically with fire frequency [16, 37], and 2) the relative growth rate of tree cover declines monotonically with tree density [38] and reaches zero at the maximum tree cover.

Obviously, fire-induced tree mortality is highly stochastic and depends on a range of factors. For instance, most tree species in the savanna biome are typically less tall [5] and better adapted to fire [39] than in the forest biome. However, as a simple mean field approximation (Fig 2B) we assume that average fire-induced losses are simply proportional to fire frequency in a fixed way. The rationale for the second assumption is that there will be density-dependent growth restriction due to crowding and competition. This is a commonly used basic assumption for models of population growth (e.g. logistic growth, generalized logistic growth and Gompertz growth). In Fig 2B we assume a linear decline in the *per-capita* growth corresponding to logistic growth, which is indeed found, for instance, in basal area growth of trees [38].

The intersection points of the growth and the loss curves represent equilibria where growth balances average loss. It can be seen that the intersection point around the threshold where loss due to fire drops, is an unstable equilibrium, as any perturbation from this specific tree cover will result in either increased tree cover towards the closed forest state, or decrease towards a very low tree cover. The existence of such an unstable equilibrium is explained by a positive feedback causing self-propagating change away from the unstable point [40, 41]. Obviously, we do not know the precise growth and loss curves. However, the observed steepness of the drop in fire occurrence implies that the results are robust in the sense that unstable points can occur at intermediate tree densities for a wide range of combinations of growth and loss curves (e.g. see S5 Fig).

## Discussion

The universality of the sharp change in tropical fire frequency around ~40% tree cover that we find is striking. Also striking is the observation that across the global tropics intermediate tree cover is systematically rare [1, 2, 5]. Our graphical model illustrates that the fire frequency pattern can explain the rarity of intermediate tree cover. The model makes it straightforward to see why this happens under a wide range of assumptions on growth curves and fire-related mortalities. In geometric terms, the reason is that the steepness of the drop in fire with increasing tree cover is unlikely to be paralleled by a similarly steep drop in growth rates around the same threshold. As a result, the growth and mortality curves tend to intersect, implying instability of intermediate tree cover. Since we derive the fire frequency directly from data, we just need to add rather standard growth equations to demonstrate that the observed bimodal patterns of tree cover are consistent with tree-cover-dependent fire as a driver.

Our simulations of the expected effects of percolation on fire frequencies illustrate the fascinating possibility that the steepness of the drop in fire frequency around a certain tree cover results from a generic fire percolation phenomenon. However, our analysis also shows that the actual tree cover at which such a percolation would happen cannot be predicted from the kind of models discussed in the literature, as the outcome depends strongly on the choice of simplifying assumptions. Nonetheless, our results do confirm that the universality of the patterns of fire and tree cover we find across the tropics are consistent with percolation as an explanation, provided that conditions such as geometry of tree distributions and their capacity to act as firebreaks are roughly universal too.

Clearly, even if the characteristics affecting percolation would be more or less invariable, there will be other relevant aspects that vary between regions. For instance, fire probabilities differ markedly between continents. The causes of those differences are still poorly understood, but may include a range of factors related to both ecology [22] and human influence [30]. There may also be less obvious aspects that cause differences between regions. For instance, mortality will depend on the susceptibility of trees to fire which is known to be dependent on their morphological traits such as bark thickness, tree size and density [42] and on allocation of biomass to roots [43]. Such traits differ from place to place and trees in fire-prone savannas have adaptations to reduce fire mortality [44]. Fire dynamics will also interact with other disturbances, particularly the effects of herbivores on grass and tree cover [45–49]. An obvious next step would now be to develop more detailed models that link the results of the long tradition of ground-based work [50] with the massive amounts of remote sensing data now available. While all remotely sensed data have important associated uncertainties [51–53], tree-cover and burned-area datasets show robust patterns over most of the tropics [52–54]. Synthesizing this information with detailed observations of fire spread and tree mortality in relation to species traits and landscape geometries in spatially explicit and ecologically realistic models may bring us closer to a true understanding of the mechanisms that shape tropical landscapes in such surprisingly universal ways.

Our inferred critical cover for tropical forest should not be confused with another possible critical cover resulting from large-scale forest-rainfall feedbacks. Forests can enhance regional rainfall, implying that a certain level of forest loss could change regional climate to a point where it becomes unfavorable for forests themselves [55–58]. Feedback between tree cover and fire acts on a local scale and is therefore independent from these regional dynamics. Nonetheless, the predicted instability of intermediate tree cover has far-reaching implications as it implies the potential for self-propagating shifts between closed forest and an open landscape when drivers such as climate change or logging reach a critical level.

## Materials and methods

### Satellite data

We used the standard MODIS burned-area product MCD45 Collection 5 [53] for the years 2002–2010 and recorded for each 500x500 m pixel whether it was burned in a given year. We did not use data from before 2002, because of a data gap in MODIS TERRA acquisitions over most of June 2001. To reduce the number of data points, we created a regularly spaced grid by using only the center pixel of each 0.1x0.1° cell, resulting in a grid of ca. 500,000 points, regularly spaced over the global tropics (latitude between 15°N and 35°S). We calculated probability of fire at 500 m scale for each tree cover class of 1% (or other variable) by counting the number of years in each class of pixels where it was recorded as burning. We excluded areas that were human-used, water or bare ground, as defined as categories [11–30 and 190–230] in the 2005 European Space Agency (ESA) Globcover dataset at 300 m resolution. Annual composite burned-area maps were generated considering the start of each year in April and the end in March the next year, coinciding with the annual global minimum fire activity during March-April [54]. The tree-cover data were extracted from the MODIS VCF Collection 5 dataset for the year 2001, before the fire-data time series [59].

We tested for climatic, topographic and anthropogenic effects on the probability of fire using twelve relevant variables. Specifically, mean annual precipitation (MAP), precipitation of the wettest quarter (PWQ) and precipitation of the driest quarter (PDQ) at 1 km resolution, which were downloaded from the WorldClim website [60]. Seasonality (MSI, Markham’s seasonality index [61]), interannual variability (coefficient of variation of MAP) and extremes (proportion of severely wet and dry years) of precipitation were calculated using the Climate Research Unit’s (CRU) monthly data at 0.5×0.5° for the period 1961‒2001 [62]. Severely wet (SPIW) or dry years (SPID) were defined as those with yearly precipitation greater than or less than 1.5 times standard deviation of long term MAP [63]. SRTM digital elevation data at 1 km resolution were downloaded from the WorldClim website. Total human population and human population in rural areas in 2005 at 0.05×0.05° were downloaded from the History Database of Global Environment (HYDE 3.1) [64] and were log-transformed. We obtained values of livestock from the FAO Gridded Livestock of the World [65]. Although this dataset uses modeling in order to extrapolate spatially, we converted the data to Tropical Livestock Units (TLU) per km^2^ where different livestock species are converted to a mean standard weight of 250 kg per individual. All spatial data were resampled to a consistent resolution of 0.1×0.1°, after which we took a sample of 1% of the data points (n = 2737). S3 Table lists the websites where the publicly available data can be downloaded.

### Minimal model of tree cover

The net change in tree cover (*T*) is modelled as the balance between the *per-capita* growth function (*g* (*T*)) and *per-capita* mortality due to forest fires (*m*(*T*)).

$$\frac{dT}{dt}=(g(T)-m(T))T$$ (1)

As growth function we use the generalized logistic growth function of Richards (growth rate *r*, yr^-1^), in which the shape of the density dependence can be adjusted by adding one extra parameter, the power *β* [66]. The carrying capacity for tree cover is implicitly set to 1 (= full cover).

$$g(T)=r(1-T^{\beta })$$ (2)

The loss due to fire is proportional to the probability of fire (*P*_*fire*_ *(T)*) to a power γ, and the average relative loss of tree cover when catching fire (*m*_*fire*_). The power γ is by default set to 1 but can be used to evaluate sensitivity to the model definition.

$$m(T)=m_{fire}P_{fire}{\left(T\right)}^{\gamma }$$ (3)

Alternatively, we assume that the relative loss of tree cover when catching fire is proportional to the tree cover:

$$m(T)=m_{fire,2}{T\;P}_{fire}{\left(T\right)}^{\gamma }$$ (4)

The annual probability of catching fire as a function of the tree cover (*P*_*fire*_ *(T)*) is determined with tropics-wide satellite data (see above). We fitted different empirical functions, using non-linear regression (lsqcurvefit in MATLAB) (Eqs 5–7) or generalized linear model fit (glmfit in MATLAB) and for logistic regression (with and without optimum) (Eqs 8 and 9):

Asymmetric optimum function ‘Double Hill function” (powers *p*_*3*_ and *p*_*5*_, half-saturation *p*_*2*_ and *p*_*4*_):

$$P_{fire}(T)=p_{1}\frac{T^{p_{3}}}{T^{p_{3}}+{p_{2}}^{p_{3}}}\frac{{p_{4}}^{p_{5}}}{T^{p_{5}}+{p_{4}}^{p_{5}}}$$ (5)

Sigmoidal Hill function (power *p*_*3*_ and half-saturation *p*_*2*_):

$$P_{fire}(T)=p_{1}\frac{T^{p_{3}}}{T^{p_{3}}+{p_{2}}^{p_{3}}}$$ (6)

Mirrored Hill function (power *p*_*3*_ and half-saturation *p*_*2*_):

$$P_{fire}(T)=p_{1}\frac{{p_{2}}^{p_{3}}}{T^{p_{3}}+{p_{2}}^{p_{3}}}$$ (7)

Standard logistic regression (parameters *p*_*1*_ and *p*_*2*_):

$$P_{fire}\;(T)=\frac{1}{1-\exp\;\left(-(p_{2}T+p_{1})\right)}$$ (8)

Logistic regression with optimum (parameters *p*_*1*,_ *p*_*2*_ and *p*_*3*_):

$$P_{fire}(T)=\frac{1}{1-\exp\;\left(-(p_{1}T^{2}+p_{2}T+p_{3})\right)}$$ (9)

These functions are not mechanistic, but are simply meant for obtaining a good fit. The parameters *p*_*1-5*_ determine the shape of the functions and are fitted using the procedure described above. We selected the most parsimonious model using the Akaike Information Criterion (AIC) assuming a binomial distribution for the fire frequency (S1 Table). We fitted the equations and did the statistics on a random sample of 1% of the points to account for spatial autocorrelation.

### Continuum and discrete percolation theory

Imagine savanna to be a very large lattice of grass. At random, a site of the lattice can be occupied by trees with a probability *p* (‘trees’) or stay unoccupied with probability (1 –*p*) (‘grass’). In the standard ‘site percolation’ framework (e.g. [26, 28]), it is assumed that fire can only travel in sites with grass by igniting neighboring grid cells with grass. However, the threshold is strongly dependent on assumptions about how cells are connected in the lattice [67] Therefore, we applied continuum percolation theory [68] to study the probability of fire as a function of tree cover.

In this approach, circles (or other shapes) are randomly distributed in a continuum of another state. We considered two possibilities: circular trees being randomly dispersed on a continuous space of grass, or circular grass patches being randomly dispersed on a continuum of trees. For computational convenience, we approximated continuum percolation by drawing overlapping circles with a radius of 20 units at random positions on a fine lattice of 1000x1000 units. We continued drawing these overlapping circles until we reached a certain tree cover. We repeated these simulations considering the continuum to be trees.

In all models, we calculated the average probability that any patch burns if a randomly chosen grass patch ignites. First, we determined the sizes of all clusters of connected grass patches *S*_*i*_. The probability that a randomly ignited cell belongs to cluster *i* is dependent on the proportion of the *N*_*g*_ grass cells that belong to that cluster (= *S*_*i*_*/N*_*g*_*)*. If this cluster is ignited, the relative area that burns is the size of the cluster divided by the total number of cells (*= S*_*i*_*/N* of the cells). Therefore, the average probability that a patch burns if any grass patch is ignited (*P*_*av*_) equals:

$$P_{av}=\sum_{i}\frac{S_{i}^{2}}{N\;N_{g}}$$ (10)

## Supporting information

S1 Fig The probability of fire as function of various variables in South America (blue circles) and Africa (red circles). This figure is not intended to be a predictive model, but we try to explain the differences in fire frequency between these continents.
We did not perform multiple regression because of covariations among variables. All variables are divided in 100 bins (except SPID and SPIW, which are discontinuous). The area of the circles indicates the frequency of observation within each bin (see legend). A. Altitude (m), B. Tree cover (%), C. Mean Annual Precipitation (MAP) (mm yr^-1^), D. Precipitation of Wettest Quarter (PWQ) (mm yr^-1^), E. Precipitation of Driest Quarter (PDQ) (mm yr^-1^), F. Coefficient of variation of annual precipitation (mm yr^-1^), G. Markham’s seasonality index (MSI) (-), H. Percentage of severely wet years (SPIW) (%), I. Percentage of severely dry years (SPID) (%), J. Livestock density in number of livestock units (km^-2^), K. Human rural population density per grid cell ^10^log (x+1) (-), L. Human population density per grid cell ^10^log (x+1) (-).
(PDF)

S2 Fig Multimodality in tree cover and the shape of the fire function match within different classes of mean annual precipitation (MAP in mm yr^-1^) for all tropics. The grayed areas approximate the range of logistic growth functions where alternative stable states are possible. a: MAP<500 mm yr^-1^; b: MAP between 500 and 1000 mm yr^-1^; c: MAP between 1000 and 1500 mm y^-1^, the maximum probability of fire here is 0.27 yr^-1^; d: MAP between 1500 and 2000 mm y^-1;^ e: MAP between 2000 and 2500 mm yr^-1^; f: MAP > 2500 mm yr^-1^.
(PDF)

S3 Fig The average probability that a grid cell (500x500 m) catches fire per year as a function of mean annual precipitation. The frequency distribution shows how often the precipitation class occurs. The lines are fitted logistic curves with optimum (for parameter values see S1 Table). A. All tropics, B. South America, C. Africa and D. Australia and Asia.
(PDF)

S4 Fig The probability of fire in a percolation model if a discrete lattice is assumed. The drop due to the percolation point is dependent on the assumptions about the connectivity between the cells. Yellow: “square grid”: fire can spread in 4 directions in the lattice, cyan: “hexagonal grid” fire can spead in 6 directions in the lattice; blue “8-neighbors” like the quare grid but fire can also spead in 4 diagonal directions.
(PDF)

S5 Fig Under a range of assumptions, each pair of growth (dashed) and loss curves (solid line) of tree cover can have three intersections. These intersection are either stable (solid circle) or unstable (open circle) equilibria. At the y-axis there is an additional unstable trivial equilibrium (open circle). In all these cases, the model has alternative stable states (see also Fig 2B). a. Different growth rates *r* (*r* = 0.0001, 0.0002 (red line), 0.0003 (cyan line), 0.0004 (purple line) and 0.0005 (yellow line)) and default loss (blue line). b. Different exponents (*β*) of the Richards’ growth curve *r* (1−*T*^*β*^) (*β* = 0.25 (green line), 0.5 (red line), 1 (cyan line), 2 (purple line) and 4 (yellow line)) and default loss (blue line). c. Different exponents (*γ*) of the relation between fire frequency and tree cover loss *P*(*T*)^*γ*^ (*γ* = 0.5 (blue line), 1 (green line), 1.5 (red line), 2 (cyan line), 2.5 (purple line) and 3 (yellow line)) and default growth (black line). d. The effect of different functions for the mortality of trees due to fire: *m*_*B*_*T*^*α*^ (α = 0 (blue line), 0.5 (green line), 1 (red line)) for two levels of growth rate (r = 0.0001 (purple line), 0.0002 (cyan line)). For other parameters see Fig 2.
(PDF)

S1 TableThe best fitting models predicting the probability of fire (500x500 m) for different variables based on the AIC.Only the six best models based on AIC are shown. **Variables:** MAP = Mean Annual Precipitation (mm yr^-1^); tree cover = tree cover (%); PWQ = Precipitation of Wettest Quarter (mm yr^-1^); PDQ = Precipitation of Driest Quarter (mm yr^-1^), std. precip. = standard deviation of annual precipitation (mm yr^-1^), cv precip. = coefficient of variation of annual precipitation (mm yr^-1^), MSI = Markham’s seasonality index (-), TLU = Livestock density in number of livestock units (km^-2^). **Area:** all = all tropic, SA = South America, AF = Africa and AU+AS = Australia and Asia. **Formula:** double Hill: $P_{fire}(T)=p_{1}\frac{T^{p_{3}}}{T^{p_{3}}+{p_{2}}^{p_{3}}}\frac{{p_{4}}^{p_{5}}}{T^{p_{5}}+{p_{4}}^{p_{5}}}$, Hill function: $P_{fire}(T)=p_{1}\frac{T^{p_{3}}}{T^{p_{3}}+{p_{2}}^{p_{3}}}$, inverse Hill function: $P_{fire}(T)=p_{1}\frac{{p_{2}}^{p_{3}}}{T^{p_{3}}+{p_{2}}^{p_{3}}}$, logistic: $Pfire\;(T)=\frac{1}{1+\exp\;\left(-(p_{2}T+p_{1})\right)}$, logistic optimum: $Pfire\;(T)=\frac{1}{1+\exp\;\left(-(p_{1}T^{2}+p_{2}T+p_{3})\right)}$. Parameters p_1_‒p_5_ differ for each of these formulas, and are simply meant to describe empirical patterns.(PDF)

S2 Table The ranges of tree cover above which the fire frequency drops (see also Fig 3). The range of the steepest drop is defined as the area where the fire frequency is between 25% and 75% of the maximum.
(PDF)

S3 Table Data sources used in this research. The data analyzed in this paper were downloaded from the following publicly available websites.
(PDF)

S1 Text Modelling the probability density of the simple model. (PDF)
