# Supplementary material for: Fire forbids fifty-fifty forest
Source: PLoS One. 2018 Jan 19;13(1):e0191027. doi: 10.1371/journal.pone.0191027 (PMC5774724; doi:10.1371/journal.pone.0191027)
Supplement: S1 Fig — This figure is not intended to be a predictive model, but we try to explain the differences in fire frequency between these continents. We did not perform multiple regression because of covariations among variables. All variables are divided in 100 bins (except SPID and SPIW, which are discontinuous). The area of the circles indicates the frequency of observation within each bin (see legend). A. Altitude (m), B. Tree cover (%), C. Mean Annual Precipitation (MAP) (mm yr-1), D. Precipitation of Wettest Quarter (PWQ) (mm yr-1), E. Precipitation of Driest Quarter (PDQ) (mm yr-1), F. Coefficient of variation of annual precipitation (mm yr-1), G. Markham’s seasonality index (MSI) (-), H. Percentage of severely wet years (SPIW) (%), I. Percentage of severely dry years (SPID) (%), J. Livestock density in number of livestock units (km-2), K. Human rural population density per grid cell 10log (x+1) (-), L. Human population density per grid cell 10log (x+1) (-). (PDF) [file pone.0191027.s001.pdf]

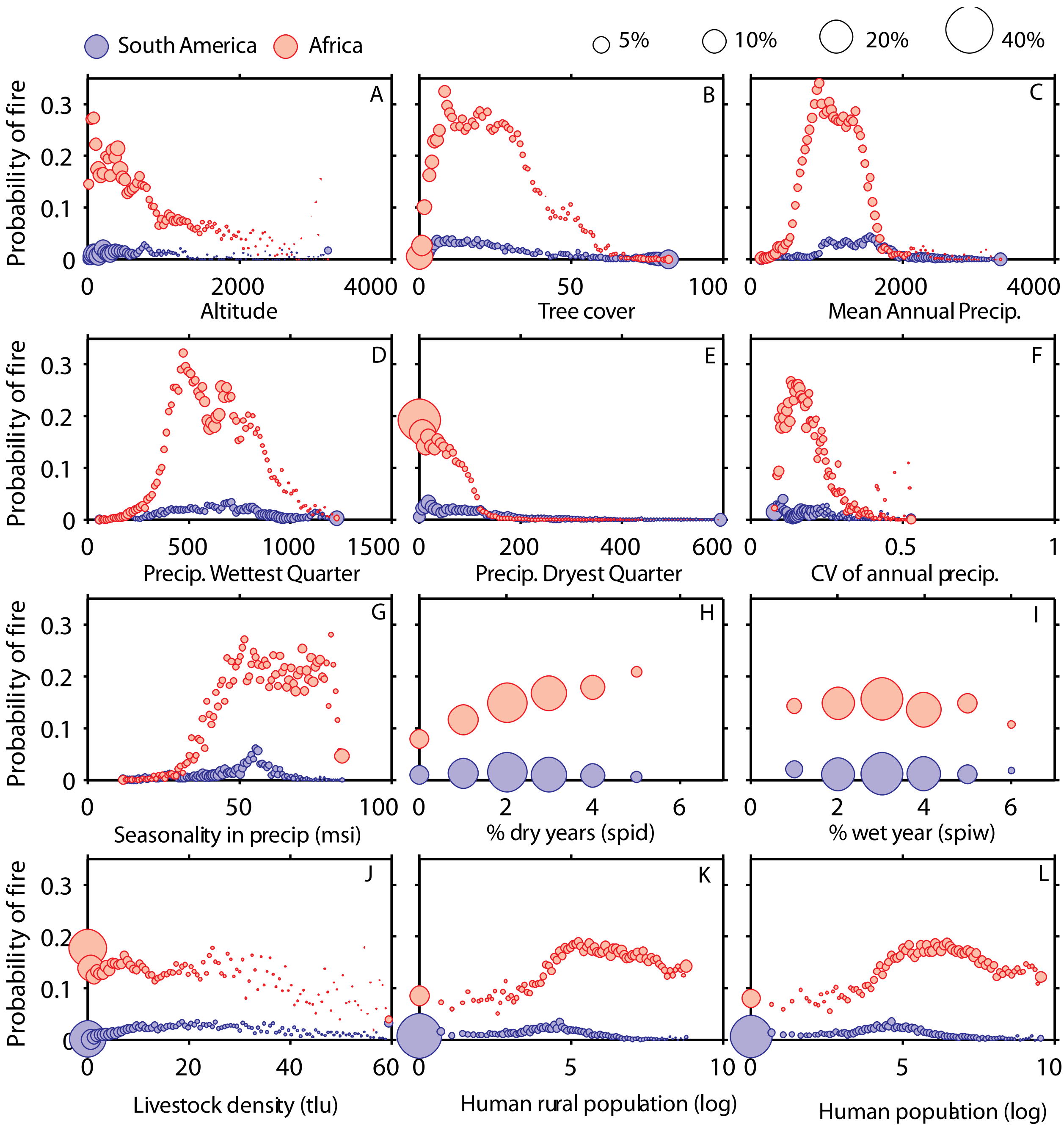

S1 Fig. The probability of fire as function of various variables in South America (blue circles) and Africa (red circles). This figure is not intended to be a predictive model, but we try to explain the differences in fire frequency between these continents.

We did not perform multiple regression because of covariations among variables. All variables are divided in 100 bins (except SPID and SPIW, which are discontinuous). The area of the circles indicates the frequency of observation within each bin (see legend). A. Altitude (m), B. Tree cover (%), C. Mean Annual Precipitation (MAP) (mm yr^{-1}), D. Precipitation of Wettest Quarter (PWQ) (mm yr^{-1}), E. Precipitation of Driest Quarter (PDQ) (mm yr^{-1}), F. Coefficient of variation of annual precipitation (mm yr^{-1}), G. Markham's seasonality index (MSI) (-), H. Percentage of severely wet years (SPIW) (%), I. Percentage of severely dry years (SPID) (%), J. Livestock density in number of livestock units (km^{-2}), K. Human rural population density per grid cell ^{10}log (x+1) (-), L. Human population density per grid cell ^{10}log (x+1) (-)
