# Supplementary material for: Fire forbids fifty-fifty forest
Source: PLoS One. 2018 Jan 19;13(1):e0191027. doi: 10.1371/journal.pone.0191027 (PMC5774724; doi:10.1371/journal.pone.0191027)
Supplement: S2 Fig — The grayed areas approximate the range of logistic growth functions where alternative stable states are possible. a: MAP<500 mm yr-1; b: MAP between 500 and 1000 mm yr-1; c: MAP between 1000 and 1500 mm y-1, the maximum probability of fire here is 0.27 yr-1; d: MAP between 1500 and 2000 mm y-1; e: MAP between 2000 and 2500 mm yr-1; f: MAP > 2500 mm yr-1. (PDF) [file pone.0191027.s002.pdf]

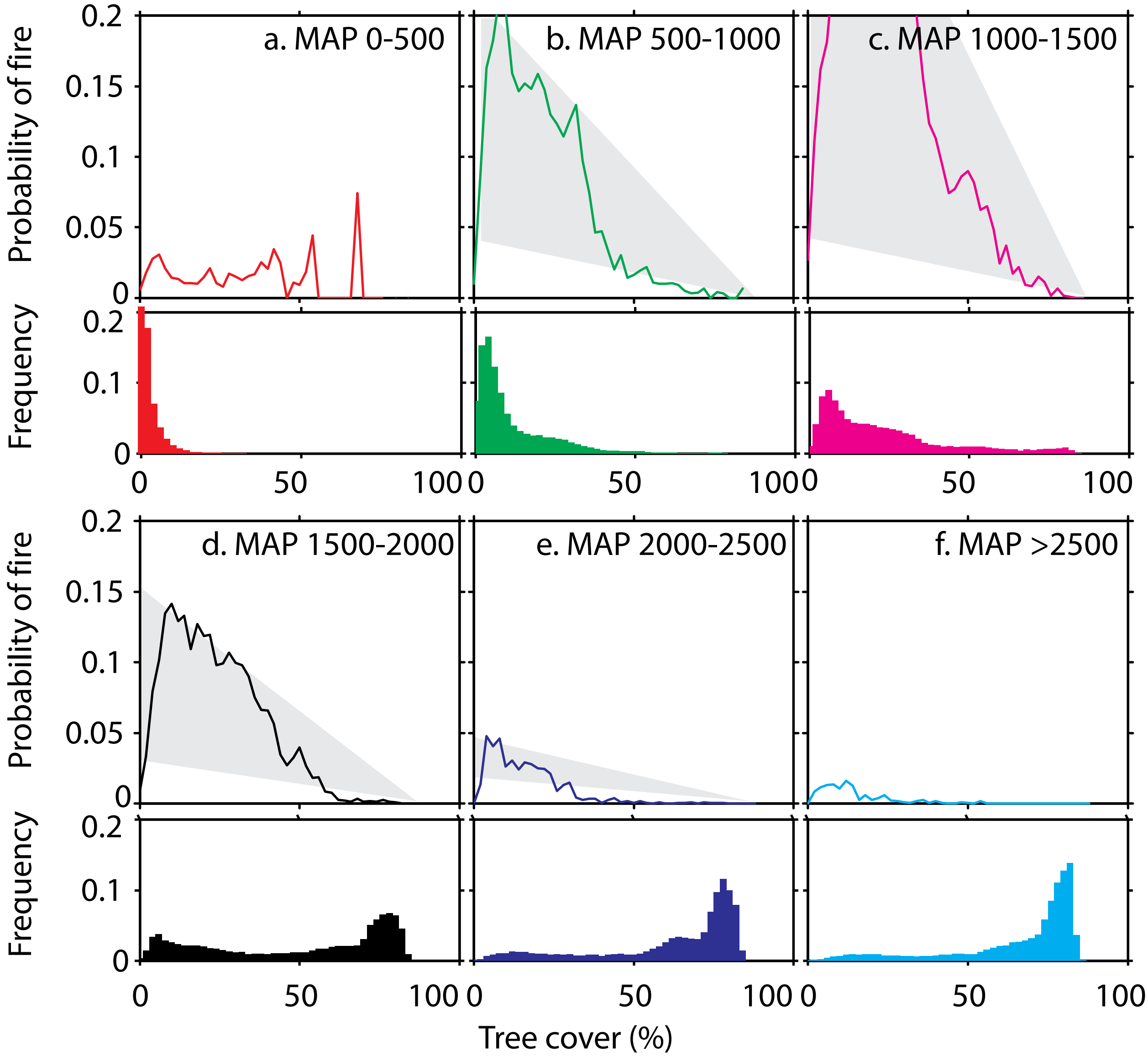

S2 Fig. Multimodality in tree cover and the shape of the fire function match within different classes of mean annual precipitation (MAP in mm yr^{-1}) for all tropics. The grayed areas approximate the range of logistic growth functions where alternative stable states are possible. a: MAP<500 mm yr^{-1}; b: MAP between 500 and 1000 mm yr^{-1}; c: MAP between 1000 and 1500 mm y^{-1}, the maximum probability of fire here is 0.27 yr^{-1}; d: MAP between 1500 and 2000 mm y$^{-1;}$ e: MAP between 2000 and 2500 mm yr^{-1}; f: MAP > 2500 mm yr^{-1}
