# Supplementary material for: Fire forbids fifty-fifty forest
Source: PLoS One. 2018 Jan 19;13(1):e0191027. doi: 10.1371/journal.pone.0191027 (PMC5774724; doi:10.1371/journal.pone.0191027)
Supplement: S3 Fig — The frequency distribution shows how often the precipitation class occurs. The lines are fitted logistic curves with optimum (for parameter values see S1 Table). A. All tropics, B. South America, C. Africa and D. Australia and Asia. (PDF) [file pone.0191027.s003.pdf]

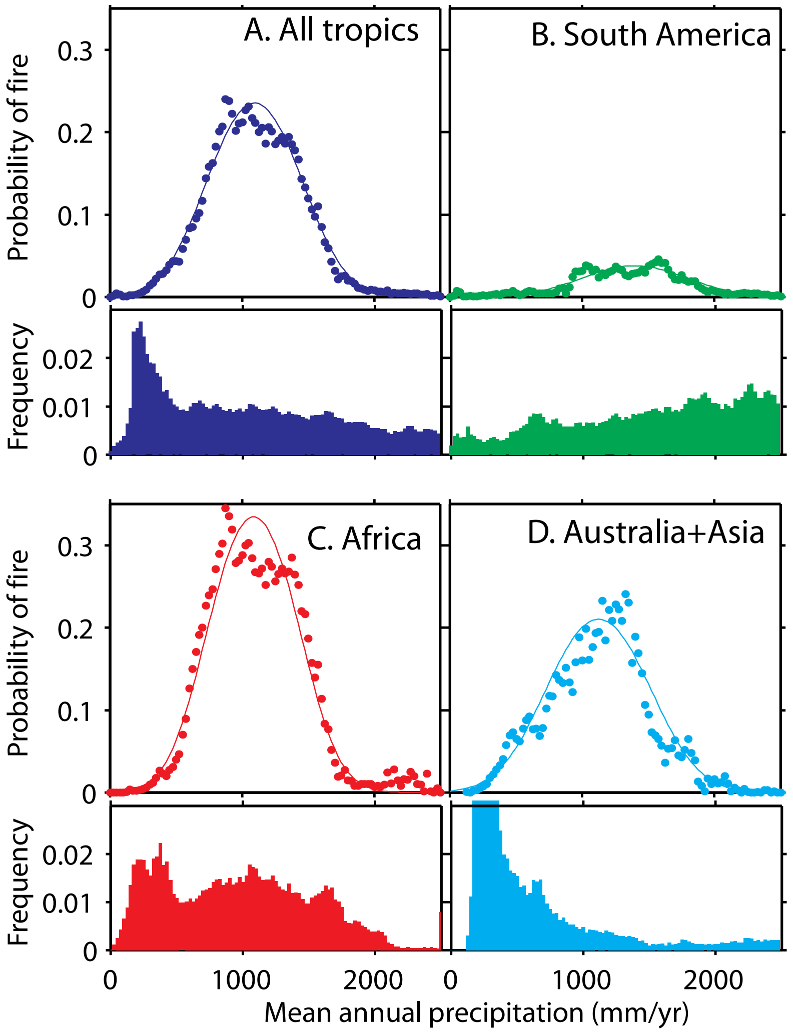

S3 Fig. The average probability that a grid cell (500x500 m) catches fire per year as a function of mean annual precipitation. The frequency distribution shows how often the precipitation class occurs. The lines are fitted logistic curves with optimum (for parameter values see Table S1). A. All tropics, B. South America, C. Africa and D. Australia and Asia.
