# Supplementary material for: Fire forbids fifty-fifty forest
Source: PLoS One. 2018 Jan 19;13(1):e0191027. doi: 10.1371/journal.pone.0191027 (PMC5774724; doi:10.1371/journal.pone.0191027)
Supplement: S4 Fig — The drop due to the percolation point is dependent on the assumptions about the connectivity between the cells. Yellow: “square grid”: fire can spread in 4 directions in the lattice, cyan: “hexagonal grid” fire can spead in 6 directions in the lattice; blue “8-neighbors” like the quare grid but fire can also spead in 4 diagonal directions. (PDF) [file pone.0191027.s004.pdf]

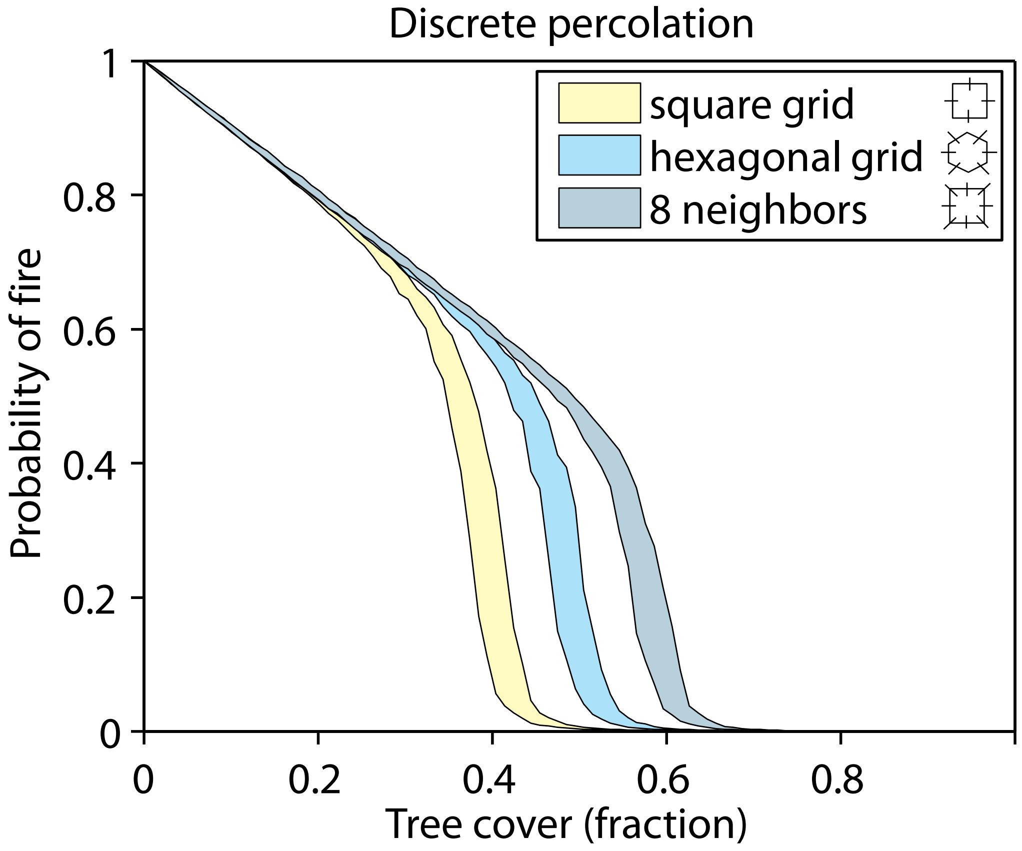

S4 Fig. The probability of fire in a percolation model if a discrete lattice is assumed. The drop due to the percolation point is dependent on the assumptions about the connectivity between the cells. Yellow: “square grid”: fire can spread in 4 directions in the lattice, cyan: “hexagonal grid” fire can spead in 6 directions in the lattice; blue “8-neighbors” like the quare grid but fire can also spead in 4 diagonal directions.
