# Supplementary material for: Fire forbids fifty-fifty forest
Source: PLoS One. 2018 Jan 19;13(1):e0191027. doi: 10.1371/journal.pone.0191027 (PMC5774724; doi:10.1371/journal.pone.0191027)
Supplement: S5 Fig — These intersection are either stable (solid circle) or unstable (open circle) equilibria. At the y-axis there is an additional unstable trivial equilibrium (open circle). In all these cases, the model has alternative stable states (see also Fig 2B). a. Different growth rates r (r = 0.0001, 0.0002 (red line), 0.0003 (cyan line), 0.0004 (purple line) and 0.0005 (yellow line)) and default loss (blue line). b. Different exponents (β) of the Richards’ growth curve r (1−Tβ) (β = 0.25 (green line), 0.5 (red line), 1 (cyan line), 2 (purple line) and 4 (yellow line)) and default loss (blue line). c. Different exponents (γ) of the relation between fire frequency and tree cover loss P(T)γ (γ = 0.5 (blue line), 1 (green line), 1.5 (red line), 2 (cyan line), 2.5 (purple line) and 3 (yellow line)) and default growth (black line). d. The effect of different functions for the mortality of trees due to fire: mBTα (α = 0 (blue line), 0.5 (green line), 1 (red line)) for two levels of growth rate (r = 0.0001 (purple line), 0.0002 (cyan line)). For other parameters see Fig 2. (PDF) [file pone.0191027.s005.pdf]

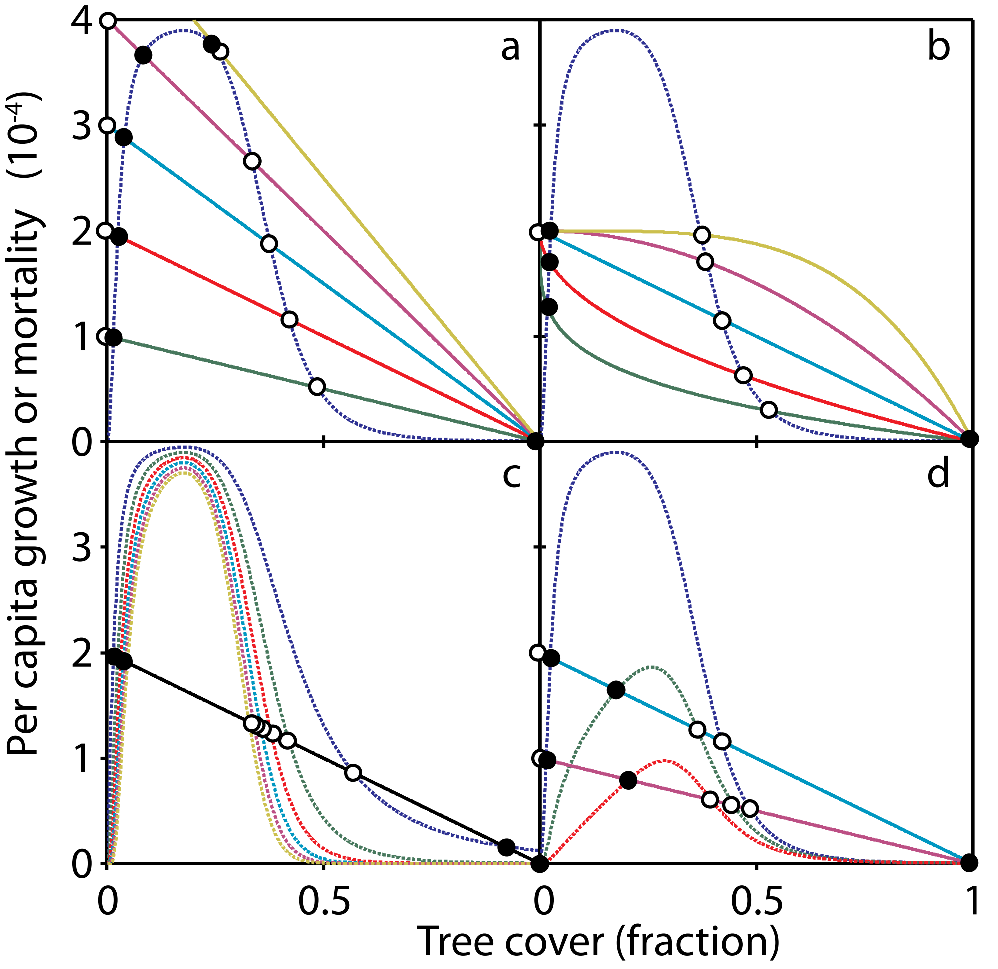

S5 Fig. Under a range of assumptions, each pair of growth (dashed) and loss curves (solid line) of tree cover can have three intersections. These intersection are either stable (solid circle) or unstable (open circle) equilibria. At the y-axis there is an additional unstable trivial equilibrium (open circle) . In all these cases, the model has alternative stable states (see also Fig 2b). a. Different growth rates r (r=0.0001, 0.0002 (red line), 0.0003 (cyan line), 0.0004 (purple line) and 0.0005 (yellow line)) and default loss (blue line). b. Different exponents (β) of the Richards' growth curve $r\ (1 - T^{\beta})$ (β = 0.25 (green line), 0.5 (red line), 1 (cyan line), 2 (purple line) and 4 (yellow line)) and default loss (blue line). c. Different exponents (γ) of the relation between fire frequency and tree cover loss $P(T)^{\gamma}$ (γ = 0.5 (blue line), 1 (green line), 1.5 (red line), 2 (cyan line), 2.5 (purple line) and 3 (yellow line)) and default growth (black line). d. The effect of different functions for the mortality of trees due to fire: $m_B T^{\alpha}$ (α = 0 (blue line), 0.5 (green line), 1 (red line)) for two levels of growth rate (r=0.0001 (purple line), 0.0002 (cyan line)). For other parameters see Fig. 2.
