# Supplementary material for: Fire forbids fifty-fifty forest
Source: PLoS One. 2018 Jan 19;13(1):e0191027. doi: 10.1371/journal.pone.0191027 (PMC5774724; doi:10.1371/journal.pone.0191027)
Supplement: S1 Table — Only the six best models based on AIC are shown. Variables: MAP = Mean Annual Precipitation (mm yr-1); tree cover = tree cover (%); PWQ = Precipitation of Wettest Quarter (mm yr-1); PDQ = Precipitation of Driest Quarter (mm yr-1), std. precip. = standard deviation of annual precipitation (mm yr-1), cv precip. = coefficient of variation of annual precipitation (mm yr-1), MSI = Markham’s seasonality index (-), TLU = Livestock density in number of livestock units (km-2). Area: all = all tropic, SA = South America, AF = Africa and AU+AS = Australia and Asia. Formula: double Hill: Pfire(T)=p1Tp3Tp3+p2p3p4p5Tp5+p4p5, Hill function: Pfire(T)=p1Tp3Tp3+p2p3, inverse Hill function: Pfire(T)=p1p2p3Tp3+p2p3, logistic: Pfire(T)=11+exp(−(p2T+p1)), logistic optimum: Pfire(T)=11+exp(−(p1T2+p2T+p3)). Parameters p1‒p5 differ for each of these formulas, and are simply meant to describe empirical patterns. (PDF) [file pone.0191027.s006.pdf]

S1 Table. The best fitting models predicting the probability of fire (500x500 m) for different variables based on the AIC. Only the six best models based on AIC are shown. **Variables:** MAP=Mean Annual Precipitation (mm yr^{-1}); tree cover=tree cover (%); PWQ=Precipitation of Wettest Quarter (mm yr^{-1}); PDQ=Precipitation of Driest Quarter (mm yr^{-1}), std. precip. =standard deviation of annual precipitation (mm yr^{-1}), cv precip. =coefficient of variation of annual precipitation (mm yr^{-1}), MSI=Markham's seasonality index (-), TLU=Livestock density in number of livestock units (km^{-2}). **Area:** all = all tropic, SA = South America, AF = Africa and AU+AS = Australia and Asia. **Formula:** double Hill: $P_{fire}(T) = p_1 \frac{T^{p_3}}{T^{p_3}+p_2^{p_3}} \frac{p_4^{p_5}}{T^{p_5}+p_4^{p_5}}$, Hill function: $P_{fire}(T) = p_1 \frac{T^{p_3}}{T^{p_3}+p_2^{p_3}}$, inverse Hill function: $P_{fire}(T) = p_1 \frac{p_2^{p_3}}{T^{p_3}+p_2^{p_3}}$, logistic: $Pfire\ (T) = \frac{1}{1+\exp(-(p_2 T + p_1))}$, logistic optimum:
$Pfire\ (T) = \frac{1}{1+\exp(-(p_1 T^2 + p_2 T + p_3))}$. Parameters p_1–p_5 differ for each of these formulas, and are simply meant to describe empirical patterns.

Variable	Area	Formula	p1	p2	p3	p4	p5	AIC
MAP	all	logistic opt.	-7.2	0.0109	-4.97E-06			10163
tree cover	all	double Hill	0.175	2.74	2.42	37.2	6.55	10596
PWQ	all	logistic opt.	-7.63	0.0208	-1.78E-05			10862
MSI	all	double Hill	0.31	67.3	3.62	82.3	114	11069
PDQ	all	inverse Hill	0.173	19.4	1.27			11138
std precip	all	logistic opt.	-4.53	0.0258	-6.90E-05			12030
tree cover	SA	double Hill	103	3.65	3.62	3.6533.4	3.56	1007
MAP	SA	logistic opt.	-7.57	0.00649	-2.48E-06			1043
std precip	SA	logistic opt.	-9.14	0.0551	-0.000133			1058
PDQ	SA	Hill function	0.02	146	-4.8			1059
TLU	SA	double Hill	2210	32.9	3.46	0.756	2.86	1073
MSI	SA	logistic opt.	-14.4	0.408	-0.00383			1077
MAP	AF	logistic opt.	-8.04	0.0135	-6.19E-06			6633
tree cover	AF	double Hill	0.282	2.66	2.39	39.7	5.57	6762
PWQ	AF	logistic opt.	-7.81	0.0221	-1.82E-05			7084
MSI	AF	double Hill	0.215	38.5	11.9	82.5	116	7232
cv precip	AF	double Hill	0.252	-0.0905	0.774	0.218	5.28	7315
std precip	AF	double Hill	3.63	127	7.31	48	2.22	7618
MSI	AU+AS	logistic	-8.8	0.0973				1676
MAP	AU+AS	logistic opt.	-6.38	0.00861	-3.73E-06			1755
std precip	AU+AS	logistic opt.	-12.1	0.0793	-1.51E-04			1772
PDQ	AU+AS	Hill function	0.193	-10.4	-1.64			1786
PWQ	AU+AS	double Hill	0.421	715	2.13	785	7.63	1845
tree cover	AU+AS	double Hill	0.117	2.66	2.48E+00	24.1	4.18	1899
