# Supplementary material for: Fire forbids fifty-fifty forest
Source: PLoS One. 2018 Jan 19;13(1):e0191027. doi: 10.1371/journal.pone.0191027 (PMC5774724; doi:10.1371/journal.pone.0191027)
Supplement: S2 Table — The range of the steepest drop is defined as the area where the fire frequency is between 25% and 75% of the maximum. (PDF) [file pone.0191027.s007.pdf]

S2 Table. The ranges of tree cover above which the fire frequency drops (see also Fig. 3). The range of the steepest drop is defined as the area where the fire frequency is between 25% and 75% of the maximum.

Continent	Tree cover where the fire frequency drops
All continents	31% to 45%
South America	24% to 45%
Africa	33% to 49%
Australia and Asia	19% to 32%
