# Supplementary material for: Fire forbids fifty-fifty forest
Source: PLoS One. 2018 Jan 19;13(1):e0191027. doi: 10.1371/journal.pone.0191027 (PMC5774724; doi:10.1371/journal.pone.0191027)
Supplement: S3 Table — The data analyzed in this paper were downloaded from the following publicly available websites. (PDF) [file pone.0191027.s008.pdf]

S3 Table. Data sources used in this research. The data analyzed in this paper were downloaded from the following publicly available websites.

Name of data base	URL	citation
MODIS tree cover product	http://glcf.umd.edu/research/portal/nasaaccess2011/vcf_index.shtml	[1]
MODIS burned area product	http://modis-fire.umd.edu/pages/BurnedArea.php	[2,3]
WorldClim data	http://www.worldclim.org/version1	[4]
CRU climate data	https://crudata.uea.ac.uk/cru/data/hrg/	[5]
HYDE human population data	http://themasites.pbl.nl/tridion/en/themasites/hyde/	[6]
Gridded livestock of the world	http://www.fao.org/ag/againfo/resources/en/glw/home.html	[7]

References

1. DiMiceli CM, Carroll ML, Sohlberg RA, Huang C, Hansen MC, et al. (2011) Annual Global Automated MODIS Vegetation Continuous Fields (MOD44B) at 250 m Spatial Resolution for Data Years Beginning Day 65, 2000 - 2010, Collection 5 Percent Tree Cover. College Park, MD, USA.: University of Maryland.
2. Roy DP, Boschetti L, Justice CO, Ju J (2008) The collection 5 MODIS burned area product - Global evaluation by comparison with the MODIS active fire product. Remote Sensing of Environment 112: 3690-3707. doi: 10.1016/j.rse.2008.05.013
3. Giglio L, Randerson JT, Van Der Werf GR (2013) Analysis of daily, monthly, and annual burned area using the fourth-generation global fire emissions database (GFED4). Journal of Geophysical Research G: Biogeosciences 118: 317-328. doi: 10.1002/jgrg.20042
4. Hijmans RJ, Cameron SE, Parra JL, Jones PG, Jarvis A (2005) Very high resolution interpolated climate surfaces for global land areas. International Journal of Climatology 25: 1965-1978. doi: 10.1002/joc.1276
5. Mitchell TD, Jones PD (2005) An improved method of constructing a database of monthly climate observations and associated high-resolution grids. International Journal of Climatology 25: 693-712. doi: 10.1002/Joc.1181

6. Klein Goldewijk K, Beusen A, Van Drecht G, De Vos M (2011) The HYDE 3.1 spatially explicit database of human-induced global land-use change over the past 12,000 years. Global Ecology and Biogeography 20: 73-86. doi: 10.1111/j.1466-8238.2010.00587.x
7. Robinson TP, William Wint GR, Conchedda G, Van Boeckel TP, Ercoli V, et al. (2014) Mapping the global distribution of livestock. PLoS ONE 9. doi: 10.1371/journal.pone.0096084
