# Supplementary material for: Fire forbids fifty-fifty forest
Source: PLoS One. 2018 Jan 19;13(1):e0191027. doi: 10.1371/journal.pone.0191027 (PMC5774724; doi:10.1371/journal.pone.0191027)
Supplement: S1 Text — (PDF) [file pone.0191027.s009.pdf]

S1 Text. Modelling the probability density of the simple model

In Fig. 2b we show a probability density function that is derived using a Fokker-Planck equation [1,2]. Here we explain this approach for non-mathematicians. Consider the following deterministic differential equation with one state variable x:

$$\frac{dx}{dt} = f(x)$$

Where *f(x)* is some function of x. The simplest way to add stochasticity is to use additive noise to the equation. This denotes random variations in the change rate of the state variable. This is done in the following way, using Itô calculus:

$$\frac{dx}{dt} = f(x) + \sigma \, dW \, dt$$

Here dW is random Gaussian noise and sigma is the standard deviation of noise. This model is stochastic, and we could run it many times to derive a probability density function. An alternative approach is to describe the probability density p(x) using a Fokker-Planck equation that is equivalent [1]. The resulting model is the following deterministic partial differential equation:.

$$\frac{\partial p(x)}{\partial t} = -\frac{\partial}{\partial x}(f(x)\, p(x)) + \frac{\partial^2}{\partial x^2}\left(\frac{\sigma^2}{2}\, p(x)\right)$$

In our case we set reflecting boundaries at a tree cover of 0 and 1(as the tree cover can never exceed these values). We start the simulation with a narrow arbitrairy initial distribution (with a surface area of 1). If we run this partial differential equation until equilibrium, the resulting distribution is independent of the initial conditions. We used the MATLAB command `pdepe` to simulate the model.

References

1. Gardiner CW (2004) Handbook of Stochastic Methods for Physics, Chemistry and the Natural Sciences. New York: Springer.
2. Grasman J, Van Herwaarden OA (2010) Asymptotic Methods for the Fokker-Planck Equation and the Exit Problem in Applications. Berlin, Heidelberg: Springer-Verlag GmbH.
